# A case of infarcted Warthin tumor misdiagnosed as Warthin-like mucoepidermoid carcinoma: diagnosis confirmed by *MAML2* rearrangement with case report and literature review

**DOI:** 10.3389/fonc.2026.1815098

**Published:** 2026-08-03

**Authors:** Yalan He, Xin Xia, Shaoce Xu, Chi Zhang

**Affiliations:** 1 Department of Stomatology, Gansu Provincial Hospital, Lanzhou, Gansu, China; 2 Department of Oral and Maxillofacial Surgery, Lanzhou University Second Hospital, Lanzhou, Gansu, China; 3 Department of Pathology, Lanzhou University Second Hospital, Lanzhou, Gansu, China

**Keywords:** case report, infarcted Warthin tumor, mastermind-like transcriptional coactivator 2 rearrangement, misdiagnosis, Warthin-like mucoepidermoid carcinoma

## Abstract

Mucoepidermoid carcinoma is a common malignant neoplasm of the parotid gland. A rare pathological subtype, Warthin-like mucoepidermoid carcinoma, closely resembles infarcted Warthin tumor in clinical presentation and histopathological features, and contributes to a high-risk of misdiagnosis. Therefore, extensive experience in morphological diagnosis by pathologists and molecular testing results are crucial for accurate diagnosis. We report a case of infarcted Warthin tumor initially misdiagnosed as Warthin-like mucoepidermoid carcinoma. The diagnosis was subsequently corrected based on molecular testing results, in conjunction with clinical features and multi-institutional pathological consultation. We report this case to raise awareness of this rare entity and to avoid inappropriate clinical management.

## Introduction

Warthin tumor (WT) occurs almost exclusively in the parotid gland and is most commonly observed in male patients with a history of smoking. Its epithelial component exhibits a characteristic bilayer arrangement, composed of an inner layer of columnar cells and an outer layer of cuboidal cells (1). The management of this tumor has become increasingly conservative, and active surveillance is now more widely accepted, particularly among older patients (2).

Warthin-like mucoepidermoid carcinoma (WL-MEC), a rare subtype of mucoepidermoid carcinoma (MEC) (3), also arises predominantly in the parotid gland but shows no clear association with smoking. Although this tumor generally has a favorable prognosis, it remains a malignant neoplasm for which a curative surgical resection is the mainstay of treatment (4).

Notably, infarcted WT (mWT) may undergo mucinous epithelial metaplasia, resulting in morphological features that substantially overlap with those of WL-MEC, thereby posing a considerable diagnostic challenge (2). Although several authors have reported this phenomenon, it remains under-recognized in the literature, potentially leading to overtreatment.

Studies have shown that the majority of MEC harbor mastermind-like transcriptional coactivator 2 (MAML2) rearrangement. Detection of this rearrangement by fluorescence *in situ* hybridization (FISH) is a specific molecular diagnostic method for this entity (5).

Here, we report a case of mWT initially misdiagnosed as WL-MEC on histopathological examination. The diagnosis was subsequently revised based on the absence of MAML2 rearrangement detected by FISH, in conjunction with clinical features, histomorphological evaluation, and multi-center pathology consultation. The patient died 6 months postoperatively, warranting a detailed report and discussion of the diagnostic and therapeutic process of this misdiagnosed case, as well as the cause of death.

## Case report

A 68-year-old male with a 50-year smoking history presented with a right infra-auricular mass that had been present for 3 years, accompanied by rapid enlargement and pain over the 6 months prior to presentation. The patient self-administered a 1-week course of cephalosporin antibiotics (details unspecified) without relief of symptoms.

Specialized physical examination revealed that the tumor was located in the lower pole of the right parotid gland. The mass demonstrated relatively well-defined borders, restricted mobility, and an absence of tenderness. Physical examination also showed cyanotic lips and a barrel chest. Oxygen saturation was 88%. Arterial blood gas analysis demonstrated a pH of 7.43, a PO_2_ of 54.7 mmHg, and a PCO_2_ of 41 mmHg.

**Figure 1:**
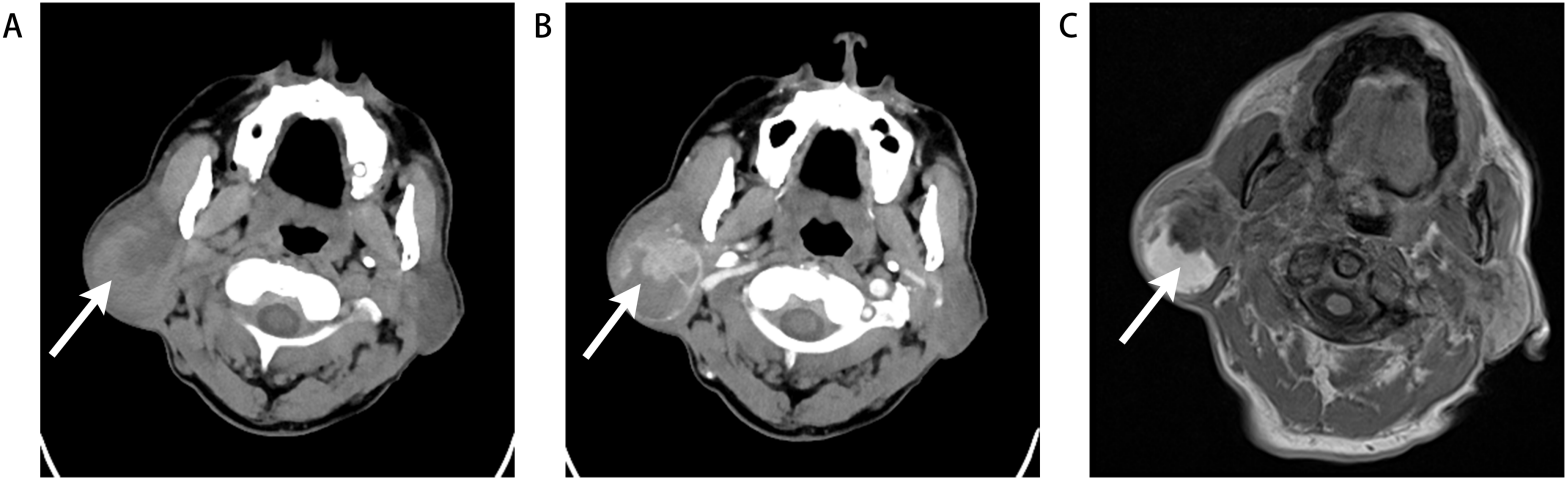
**(A)** Non-contrast neck computed tomography (CT) imaging shows a round, mixed-density mass (arrow) in the superficial lobe of the right parotid gland, containing hyperdense hemorrhagic foci and relatively hypodense solid components. **(B)** Contrast-enhanced neck CT demonstrates marked enhancement of the solid components, internal septations and a capsule, multiple small vessels surrounding and within the mass, and non-enhancing hemorrhagic/necrotic/cystic areas. **(C)** Non-contrast neck magnetic resonance imaging (MRI), axial T1-weighted images reveals a heterogeneous-signal mass in the right parotid gland, with solid components showing iso-to slightly hypointense signals and hemorrhagic/necrotic/cystic areas appearing hyperintense.

Imaging studies revealed a well-defined, round mass measuring 4.49×4.6 cm in the posterior inferior portion of the superficial lobe of the right parotid gland. The mass exhibited necrotic-cystic changes and hemorrhage (**Figure 1**). Fine-needle aspiration biopsy (FNAB) revealed scattered clusters of atypical cells within abundant mucinous material, with cells exhibiting mucin-rich cytoplasm, suggesting a low-grade malignant tumor of the parotid gland. However, the specific histological subtype could not be determined. Cardiac ultrasonography demonstrated mild-to-moderate regurgitation of the tricuspid and aortic valves. Pulmonary function tests indicated moderate-to-severe ventilatory dysfunction. Chest computed tomography (CT) revealed chronic bronchitis, bilateral interstitial lung changes, and mild widening of the pulmonary trunk.

The multidisciplinary team (MDT) from the Departments of Critical Care Medicine, Respiratory Medicine, Cardiovascular Medicine, Nutrition, and Pathology diagnosed the patient with moderate chronic obstructive pulmonary disease (COPD) and pulmonary hypertension. The MDT recommended minimizing surgical duration and trauma and implementing early extubation and oxygen therapy postoperatively.

Considering the patient’s advanced age, 50-year smoking history, and severe underlying comorbidities including moderate COPD and pulmonary hypertension, the surgical team formulated a surgical plan based on the MDT recommendations and preoperative ancillary examination results. The planned procedure was right partial parotidectomy combined with tumor resection and sentinel lymph node biopsy. The resected specimen appeared grayish-white and grayish-red with an intact capsule. The cut surface was cystic, containing coffee-colored gelatinous material. Two grayish-white, cauliflower-like solid tumors were observed within the cystic cavity. Intraoperative frozen section results indicated focal atypia in the tumor cells, with the definitive diagnosis pending final pathology. The submitted sentinel lymph nodes showed no evidence of carcinoma metastasis. Postoperatively, the medical team actively provided the patient with oxygen therapy, sputum expectoration, infection prophylaxis, nutritional support, and thrombosis prevention while closely monitoring vital signs.

**Figure 2:**
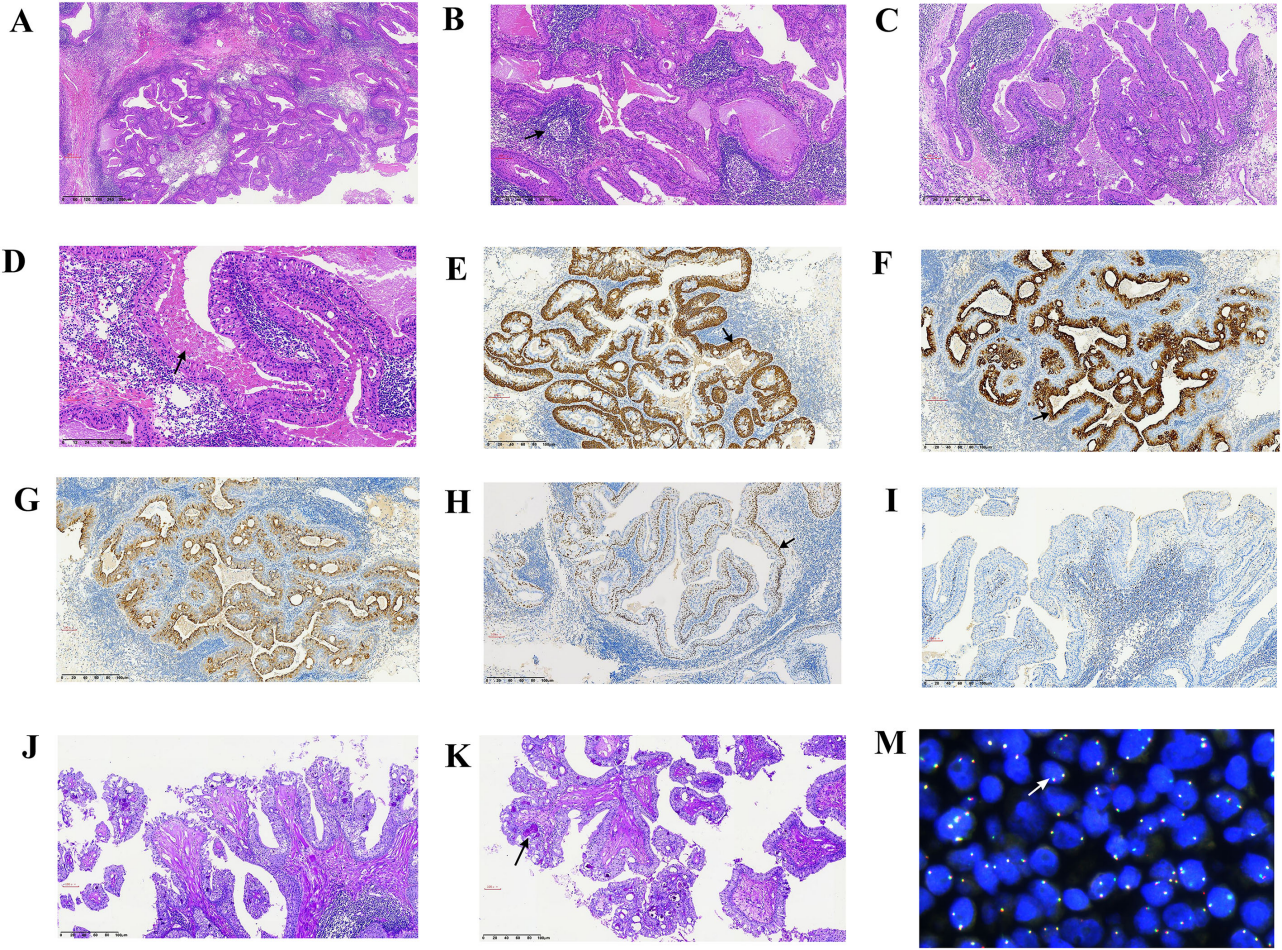
On hematoxylin and eosin (HE) staining, the tumor epithelium exhibits a papillary hyperplastic pattern. **(A, B)** Abundant lymphocytic stroma is observed (black arrows). **(C, D)** Focal areas show crowded, densely packed epithelial cells with surface necrosis (black arrows); however, the classic bilayer cellular arrangement of WT is still visible (white arrows). **(E–I)** Immunohistochemical staining results [**(E)** CK5/6; **(F)** CK7; **(G)** CK8/18; **(H)** p63; **(I)** Ki-67]: CK7 shows luminal membrane positivity; CK5/6 and p63 show basal cell positivity (black arrows). **(J–K)** Alcian blue-periodic acid–Schiff staining: Specifically highlights mucinous cells (black arrows). **(M)** Fluorescence *in situ* hybridization analysis shows no MAML2 gene rearrangement in tumor cells (white arrow).

The pathology report indicated multicystic tumor growth with abundant lymphoid tissue in the interstitium. The neoplastic epithelium was composed of oncocytic cells, clear cells, and mucinous cells. The tumor exhibited a multilayered or papillary growth pattern projecting into the cystic lumen. The epithelial cell nuclei were round, oval, or vesicular with visible nucleoli, rare mitotic figures, and no significant atypia. The tumor involved the adjacent salivary glands and was closely associated with vascular structures but showed no invasion (**Figure 2**). Seven lymph nodes from the perisalivary and upper deep cervical regions showed reactive hyperplasia with no tumor cells. The surgical margins were negative. The tumor size was 4×3×2 cm. The pathological morphology was consistent with WL-MEC, low-grade, staged as T2N0M0 (AJCC 8th Edition, 2017) (4).

Given that the clinical features of this case were inconsistent with WL-MEC and more closely resembled WT, our team invited specialized pathologists from multiple centers for review and performed FISH testing for MAML2 rearrangement. The consultation opinion concluded that microscopic examination of the right parotid gland revealed multilayered and papillary growth of oncocytic tumor epithelium containing mucinous and clear cells, with abundant interstitial lymphocytes. The FISH results (no MAML2 rearrangement) (**Figure 2M**) supported the diagnosis of mWT. Consequently, our team revised the diagnosis to right parotid WT and provided postoperative rehabilitation and follow-up guidance to the patient.

The patient had long lived alone and exhibited extremely poor compliance, creating significant difficulties for follow-up. During a telephone follow-up by our team 3 months after surgery, the patient reported satisfactory wound healing and no abnormal symptoms but admitted to not following medical advice regarding smoking cessation or home oxygen therapy. At the 6-month postoperative follow-up, the patient’s family informed us of his death, noting that symptoms of chest pain and syncope had occurred 1 day prior to death without timely medical intervention. Considering the patient’s pre-existing conditions, including COPD, pulmonary hypertension, and structural cardiac changes, coupled with the symptoms before death, we suspect that the patient may have died from a cardiovascular event. Regrettably, due to the absence of an autopsy and the lack of available medical records, the exact cause of death cannot be definitively established.

A timeline concisely showing the medical course of this unusual case is presented in **Table 1**.

**Table 1:** Timeline table of case report.

Time point	Clinical event summary	Diagnostic test results	Treatment/intervention	Outcome/notes
2021	Right infra-auricular mass noted; asymptomatic	NA	NA	Did not seek medical attention
April 2024	Rapid enlargement of the mass with pain; self-administered oral cephalosporins for 1 week, but no response	NA	NA	Symptom progression
October 2024 (initial presentation)	Physical examination: mass in lower pole of the right parotid gland (well-defined borders, restricted mobility, non-tender); cyanotic lips, barrel chest, SpO_2_ 88% Arterial blood gas: pH 7.43; PO_2_ 54.7 mmHg; PCO_2_ 41 mmHg	Imaging: 4.49×4.6 cm round mass in the right parotid gland with necrotic-cystic changes and hemorrhage Echocardiography: mild-to-moderate tricuspid and aortic regurgitation Pulmonary function tests: moderate-to-severe ventilatory dysfunction Chest CT: chronic bronchitis, bilateral interstitial lung changes, mild widening of pulmonary trunk FNAB: low-grade malignant tumor, specific subtype undetermined	NA	Diagnosed with moderate COPD and pulmonary hypertension
October 2024 (MDT meeting)	Multidisciplinary team consultation (critical care, respiratory medicine, cardiology, nutrition, pathology)	NA	Decision: minimize surgical duration and trauma; perform partial parotidectomy and sentinel lymph node biopsy	Individualized conservative surgical strategy
Next day	Uncomplicated surgery; resection margin 1–2 cm from tumor, facial nerve dissected and preserved	Frozen section: focal atypia, negative margins, no lymph node metastasis	Right parotid tumor resection, partial gland excision, and sentinel lymph node biopsy	Successful surgery
Several days postoperatively	NA	Gross specimen: intact capsule, cystic cut surface containing brown gelatinous material and two grayish-white cauliflower-like solid nodules Histopathology: multicystic growth with abundant lymphoid stroma; tumor cells composed of oncocytic, clear, and mucinous cells; round/oval/vesicular nuclei with visible nucleoli, rare mitotic figures, no significant atypia; involvement of adjacent salivary glands without invasion; seven lymph nodes negative; negative margins; size 4×3×2 cm Immunohistochemistry: CK7 (luminal+); CK5/6, p63 (basal+) Initial diagnosis: WL-MEC, low-grade, T2N0M0 (AJCC 8th ed.)	NA	Clinical-pathological discordance (clinical features more consistent with WT)
Review stage	Multi-center pathology expert consultation	Microscopic examination: multilayered and papillary growth of oncocytic tumor epithelium containing scattered mucinous and clear cells with abundant interstitial lymphocytes FISH: no MAML2 rearrangement	NA	Diagnosis revised: mWT
January 2025 (3 months postoperatively)	Telephone follow-up: satisfactory wound healing, no abnormal symptoms	NA	NA	Poor compliance: continued smoking, did not adhere to home oxygen therapy
April 2025 (6 months postoperatively)	Death reported by family; chest pain and syncope 1 day prior to death, without timely medical intervention	NA		Presumed cardiovascular event (autopsy not performed, exact cause of death unknown)

FNAB, fine-needle aspiration biopsy; IHC, immunohistochemistry; CK, cytokeratin; COPD, chronic obstructive pulmonary disease; MDT, multidisciplinary team; SLNB, sentinel lymph node biopsy; FISH, fluorescence in situ hybridization; AJCC, American Joint Committee on Cancer; SpO_2_, oxygen saturation; PO_2_, partial pressure of oxygen; PCO_2_, partial pressure of carbon dioxide; NA, not available.

## Discussion

WT with CRTC1-MAML2 fusion positivity and histopathological features more consistent with MEC was first designated as WL-MEC by Ishibashi et al. (5) The CRTC1-MAML2 fusion gene is a major driver of MEC and plays a decisive role in the development and maintenance of this disease (6). It regulates gene expression through CREB-dependent and CREB-independent mechanisms, thereby participating in key cellular processes including growth, proliferation, survival, migration, and metabolism of MEC cells (7).

WL-MEC shows a female predilection, a wide age range, and a peak incidence in the 50–59 years age group. Most cases arise in the parotid gland and present as an indolent nodular mass that is often asymptomatic (5). Conversely, WT is typically characterized by a slight male predominance and an association with smoking, with patients being significantly older than those with WL-MEC (8).

WL-MEC typically exhibits low-grade histological features. Microscopic examination reveals a multicystic architecture with abundant lymphoid stroma. The cysts are lined by a single layer, disordered bilayer, or multilayered epithelium of oncocytic cells, which are flattened with vesicular nuclei. Focal areas may contain classical MEC components. In some cases, extensive stromal hyalinosis and mucous extravasation can be observed (9). mWT frequently demonstrates squamous and mucous epithelial metaplasia, resulting in significant morphological overlap with WL-MEC and a substantial risk of misdiagnosis (10). However, experienced pathologists can effectively distinguish between the two entities based on the following histomorphological features: In mWT, focal areas still retain the characteristic bilayer oncocytic epithelial architecture (even in extensively metaplastic regions), whereas WL-MEC completely lacks this feature. Mucous metaplasia in mWT is typically confined to necrotic areas or regions adjacent to inflammatory stimuli, with little mucous change observed elsewhere in the tumor. Furthermore, mWT does not exhibit true invasive behavior (8). Avoiding misdiagnosis of multilayered oncocytic mWT lacking solid epidermoid cell nests as WL-MEC is critical; the presence of ductal structures is a key diagnostic point for mWT. Histopathological examination of the present case revealed florid cellular proliferation in focal areas (suggestive of hyperproliferative activity with malignant potential), forming solid cell nests accompanied by nuclear atypia. Atypical mucin-filled cells were scattered among these nests. Based solely on local hematoxylin and eosin (HE) staining, these features closely mimicked low-grade MEC. However, upon careful observation, the classic oncocytic epithelial bilayer architecture could still be identified in the HE-stained sections (white arrow in **Figure 2C**).

In addition to histomorphology, immunohistochemical findings also provide certain value for differential diagnosis, although specific immunohistochemical markers that reliably distinguish WL-MEC from WT remain scarce. WL-MEC and mWT express markers associated with luminal differentiation (e.g., CK7, CK8, and CK18) as well as markers related to squamous and basal cell differentiation (e.g., CK5/6, p40, and p63). Differences lie in the staining pattern: CK7 shows predominantly luminal membrane positivity in mWT, localized to the luminal oncocytic/columnar cells, whereas it exhibits diffuse positivity in WL-MEC. CK5/6, CK14, and p63 show specific positivity in the basal cells of mWT but show diffuse positivity in WL-MEC. Mitochondrial antigen is strongly positive in luminal oncocytic cells of mWT and diffusely positive in WL-MEC (11). BSND has recently been identified as a potentially useful diagnostic marker for distinguishing WT from WL-MEC. Using a cut-off of ≥10% BSND immunopositivity effectively discriminates between the two entities: BSND shows patchy positivity in WT, whereas WL-MEC is negative (12). However, given the limited sample size of current studies, the reliability of this marker requires confirmation through large-scale investigations. In the present case, the tumor epithelium showed widespread CK7 expression with a luminal membrane pattern (black arrow in **Figure 2F**), whereas basal cells predominantly expressed CK5/6 (black arrow in **Figure 2E**) and p63 (black arrow in **Figure 2H**), a staining pattern more consistent with WT.

However, it is important to note that among pathologists with limited exposure to head and neck tumor specimens or without subspecialty experience, diagnosis based solely on histopathological observation carries a high-risk of misdiagnosis, rendering molecular testing particularly necessary. MAML2 rearrangement is detected in the majority of MECs and, in addition, can also be found in thymic tumors (types B2/B3), mWT, and hidradenoma (13, 14). Therefore, considering the specific anatomical site and tissue of origin, whether MAML2 rearrangement is specific for MEC in parotid gland tumors remains controversial. This controversy primarily centers on whether WT of the parotid gland harbors this translocation in addition to MEC. As multiple studies have confirmed the absence of MAML2 rearrangement in WT (8, 10, 15), previously reported cases of WT positive for MAML2 rearrangement are more likely attributable to histological misdiagnosis or the coexistence of WT and MEC (16). Consequently, MAML2 rearrangement is highly supportive for the diagnosis of parotid MEC. According to the literature, the detection rate of MAML2 rearrangement in parotid MEC ranges from ~59%–88% (17–19), and approaches 100% in pediatric and adolescent cases (20).

As a histological subtype of MEC, WL-MEC generally exhibits an indolent clinicopathological course and low aggressive biological behavior. Surgical resection achieves favorable outcomes in most patients with WL-MEC, with a 5-year overall survival rate of 97.0% (10). A systematic review of 78 WL-MEC cases conducted by de Oliveira et al. found that all WL-MECs harbored MAML2 rearrangement (15). Although clinical studies on WL-MEC remain scarce, and its specific clinical behavior, histological and genomic features have not been fully characterized, the reported detection rate of MAML2 rearrangement in WL-MEC across existing studies is 100% (**Table 2**) (8–10, 21–26), with no negative reports to date. Such a high detection rate suggests that MAML2 rearrangement may represent a unique molecular signature of salivary WL-MEC and could serve as robust evidence for the diagnosis of this entity. However, whether it can be regarded as the gold standard for the diagnosis of WL-MEC requires further validation through extensive clinical and basic research. Therefore, in-depth investigation of the specific clinical, histological and genomic features of WL-MEC is warranted to facilitate accurate diagnosis and appropriate treatment.

**Table 2:** Cases of WL-MEC by MAML2 break-apart FISH.

Author (year)	Cases (n)	Age (years)	Sex (F/M)	Smoking history	Location	MAML2 rearrangement	Low-grade
Wang, 2024 (10)	29	8–68	19F/10M	N (27/29)	Parotid gland (29/29)	Positive (29/29)	Y
Bieńkowski, 2020 (8)	2	30	F	Y	Parotid gland	Positive	Y
		51	F	NA	Parotid gland	Positive	Y
Yan, 2023 (9)	1	41	F	NA	Parotid gland	Positive	NA
Zhang, 2019 (21)	1	36	M	No	Parotid gland	Positive	Y
Zhang, 2021 (22)	9	10-75	5F/4M	NA	Parotid gland (9/9)	Positive (9/9)	Y
Basak, 2022 (23)	3	16	F	NA	Parotid gland	Positive	Y
		27	M	NA	Parotid gland	Positive	Y
		53	M	NA	Parotid gland	Positive	Y
Yang, 2024 (24)	2	60	F	No	Parotid gland	Positive	NA
		29	M	No	Submandibular gland	Positive	NA
Hang, 2017 (25)	2	53	F	NA	Parotid gland	Positive	NA
		53	F	Y	Parotid gland	Positive	NA
Noda, 2022 (26)	1	16	F	NA	Parotid gland	Positive	NA

F, female; M, male; NA, not available; Y, yes. All cases were confirmed by MAML2 break-apart FISH. Data in parentheses indicate the number of cases with the feature relative to the total number of cases in that study (e.g., 27/29 indicates 27 out of 29 cases).

Given the important diagnostic value of MAML2 rearrangement in WL-MEC, accurate and reliable detection methods are essential. Although fluorescence *in situ* hybridization (FISH) has limitations, including the ability to analyze only limited tissue areas and the difficulty of precisely correlating FISH results with extensive histological features, previous studies have validated the accuracy of FISH for detecting MAML2 rearrangement using reverse transcription polymerase chain reaction (RT-PCR). Moreover, compared with RT-PCR, FISH can detect all types of MAML2 translocation and is more reliable on formalin-fixed, paraffin-embedded (FFPE) tissue. Detection of MAML2 gene break-apart using FISH is the preferred method in clinical practice (8, 27). When mWT exhibits mucous and squamous metaplasia, rendering histopathological diagnosis difficult, FISH detection of MAML2 rearrangement, in conjunction with anatomical site and histopathological observation, is a critical differential diagnostic tool (5, 28).

In terms of specimen selection, although FNAB serves as a first-line minimally invasive sampling method for evaluating salivary gland tumors and has a high accuracy rate (81%–100%) for distinguishing benign from malignant lesions, its accuracy for specific subtyping of salivary gland tumors varies considerably (48%–94%) (29). Moreover, mWT often presents with an atypical squamous or mucinous background that can easily be confused with MEC, and FNAB is unable to assess tissue architecture or invasive features. These factors limit its utility in the differential diagnosis between mWT and WL-MEC. Therefore, relying solely on FNAB results carries a higher risk of misdiagnosis in this context. In the present case, the failure to achieve a definitive diagnosis by FNAB may be attributed to the presence of scattered atypical cell clusters within abundant mucinous material (with some cells showing mucin-rich cytoplasm), combined with infarction-induced cellular degeneration, loss of architectural information, and insufficient tissue quantity for evaluating invasive features. When clinical and radiological findings suggest that a parotid tumor may be WT or WL-MEC, particularly when FNAB results are atypical or inconsistent with clinical features, obtaining a surgical resection specimen for complete histopathological evaluation, combined with MAML2 FISH testing, should be considered to establish a definitive diagnosis. Surgical resection specimens not only provide sufficient tissue quantity for FISH analysis but also enable precise correlation between extensive histological observation and molecular testing results, representing the ‘gold standard’ specimen type for differential diagnosis.

WL-MEC and mWT demonstrate high histopathological similarity. Misdiagnosis of the former as the latter is relatively common, whereas the reverse misdiagnosis is rare. Our team reports a case of mWT initially misdiagnosed as WL-MEC, with the diagnosis subsequently revised based on clinicopathological features, consultation opinions from external pathology centers, and a negative MAML2 FISH result. Our team proposes that for challenging cases, accurate differentiation between these two entities requires comprehensive evaluation of clinical characteristics, morphology, immunohistochemical expression patterns (CK7, p63/CK5/6, BSND), and MAML2 molecular testing results (**Table 3**), and should not rely solely on histomorphology. In resource-limited settings or primary hospitals, clinicians should fully recognize the limitations of relying solely on histopathological consultation. When the diagnosis is uncertain, treatment decisions should be made cautiously to avoid overtreatment. The use of molecular testing and multi-center pathology consultation is recommended to establish a definitive diagnosis; however, these approaches are often time-consuming and may delay treatment decisions. Furthermore, for older aged patients with head and neck tumors with concomitant systemic diseases, MDT management, post-discharge follow-up planning, and patient compliance should be emphasized to improve treatment outcomes.

**Table 3:** Differential diagnosis between Warthin-like mucoepidermoid carcinoma and infarcted Warthin tumor.

Feature	mWT	WL-MEC
Bilayered oncocytic epithelium	Present	Absent
MAML2 rearrangement	Negative	Positive
CK7 pattern	Luminal	Diffuse
CK5/6 & p63	Basal	Diffuse
Invasion	Absent	Present

CK, cytokeratin; BSND, barttin CLCNK type accessory subunit beta (a marker for oncocytic differentiation); MAML2, mastermind-like transcriptional coactivator 2; WL-MEC, Warthin-like mucoepidermoid carcinoma; WT, Warthin tumor; Mwt, infarcted Warthin tumor. For BSND, a cutoff of ≥10% positive tumor cells is considered positive. For MAML2 rearrangement, break-apart FISH is the recommended detection method.

The patient had an uneventful postoperative recovery with no evidence of local recurrence or distant metastasis. Consequently, the patient’s death was considered unrelated to tumor progression. Although a cardiovascular event was considered a possible cause of death, it should be noted that this assumption remains speculative in the absence of an autopsy or complete medical records. Therefore, the definitive cause of death could not be established. This single-case study has clear limitations, and the generalizability of our conclusions is limited, requiring validation in larger future studies.

## Patient perspective

Despite our best efforts, a formal patient perspective statement could not be obtained directly from the patient, as the patient is deceased and lived alone for many years without any direct next of kin. However, during the 3-month postoperative telephone follow-up, the patient reported that the wound had healed well, with no pain, swelling, or other discomfort in the surgical area, and that he was able to carry out daily activities independently and was satisfied with the surgical outcome. At the 6-month follow-up, information obtained from a neighbor confirmed that the patient had remained free of significant discomfort in the surgical area throughout the postoperative period and that his daily life had not been notably affected. We acknowledge the absence of a direct patient perspective statement as a limitation of this report.

## Data Availability

The original contributions presented in the study are included in the article/supplementary material. Further inquiries can be directed to the corresponding author.
